# DeiT-MLP-mixer for the preoperative prediction of axillary lymph node involvement in breast cancer via ultrasound imaging

**DOI:** 10.1186/s12880-026-02256-9

**Published:** 2026-04-06

**Authors:** Nazeh Asadoorian, Shokufeh Yaraghi, Araeek Tahmasian, Rasoul Fatehifard, Maryam Rezazadegan, Narjes Mottaghi

**Affiliations:** 1 Department of Computer Engineering, Shahid Ashrafi Esfahani University, Isfahan, Iran; 2 Health Management and Economics Research Center, Isfahan University of Medical Sciences, Isfahan, Iran; 3 Najafabad University of Medical Sciences, Isfahan, Iran

**Keywords:** Data-efficient image transformer (DeiT), MLP-mixer, Axillary lymph node (ALN) involvement, Deep learning, Vision transformer (ViT), Breast ultrasound

## Abstract

**Background:**

Accurate preoperative prediction of axillary lymph node (ALN) metastasis is essential for guiding treatment decisions in breast cancer patients and avoiding unnecessary surgeries. Conventional methods, including clinical examination and imaging, often lack sufficient accuracy, whereas standard surgical procedures such as sentinel lymph node biopsy and axillary lymph node dissection (ALND) are invasive and carry potential complications. This study aims to develop a reliable, noninvasive deep learning model for predicting ALN status via grayscale ultrasound images.

**Methods:**

We propose a hybrid deep learning architecture, DeiT-MLP-Mixer, which combines a data-efficient image transformer (DeiT) for global feature extraction with an MLP-Mixer-inspired classifier for final prediction. The DeiT module captures global spatial relationships, whereas the MLP-Mixer integrates local and global features through spatial and channel mixing. The model was trained and validated on a real-world dataset of 502 breast cancer patients (1,506 augmented ultrasound images), with ALN status confirmed via postoperative pathology. The performance was evaluated in terms of accuracy, precision, recall, specificity, F1 score, and loss. Compared with pretrained convolutional neural networks (CNNs) and standard transformer-based models, the proposed approach has improved accuracy and reliability, particularly in handling limited clinical datasets.

**Results:**

The DeiT-MLP-Mixer achieved high performance on the test set, with an accuracy of 95.70%, precision of 0.9760, recall of 0.9242, specificity of 0.9867, F1 score of 0.9494, and a loss of 0.1345. It outperformed baseline models, including Vision Transformer, DeiT, and pretrained CNNs such as InceptionV3, MobileNetV2, VGG16, ConvNext, EfficientNet, and DenseNet.

**Conclusions:**

Our study presents the DeiT-MLP-Mixer as a practical and effective technique for predicting ALN status in breast cancer patients, demonstrating its effectiveness in achieving higher accuracy and specificity than conventional CNNs and standard transformer models do, even with limited clinical data. This work contributes to ongoing efforts to use artificial intelligence in the development of noninvasive tools that support clinicians in preoperative decision-making. In particular, knowing the presence or absence of ALN involvement prior to surgical planning can facilitate earlier prognostic assessment, guide the subsequent treatment course, and help reduce unnecessary invasive axillary surgical procedures.

## Introduction

Breast cancer remains the most frequently diagnosed cancer in women globally and the second leading cause of cancer death after lung cancer [1]. Metastasis can spread cancer cells via lymph nodes or blood vessels with axillary lymph nodes (ALNs), which are among the most common routes of spread [2, 3]. ALN involvement is a key factor in staging, treatment planning, and prognosis. It is also a crucial resource for postoperative chemotherapy and radiotherapy decisions. Before surgery, it is important to choose an accurate and secure diagnosis of ALNs, which can help avoid unnecessary axillary surgery and postoperative complications such as lymphedema, sensory discomfort, and restricted upper limb movement [4–6]. Currently, Axillary lymph node dissection (ALND) and sentinel lymph node biopsy (SLNB) are the standard approaches, but both are invasive and may cause postoperative complications [7]. Up to 70% of early-stage breast cancer patients do not have ALN metastasis. Surgery in these patients can lead to overtreatment due to side effects such as discomfort, arm lymphedema, restricted shoulder movement, and numbness [8]. SLNB has therefore replaced ALND in most cases, as it reduces the need for unnecessary dissection while still providing accurate information to guide treatment decisions [9–11].

Since both surgical approaches are aggressive and may cause complications, a reliable, noninvasive method for predicting ALN status is needed to avoid unnecessary surgery in ALN-negative patients. Clinical examination remains the most common approach, but its accuracy strongly depends on the physician’s experience. Imaging techniques such as ultrasound, mammography, CT, and MRI can improve detection, but their performance is still limited, with clinical exam sensitivity as low as 30% and combined imaging often missing a substantial portion of metastases [12, 13].

Ultrasound is widely used for the preoperative staging, diagnosis, and screening of breast cancer. In particular, axillary ultrasound is considered the most practical and safest noninvasive method for evaluating ALN status before surgery [14, 15]. Additionally, to support preoperative planning, ALNs with suspicious morphology are often evaluated via needle biopsy guided by ultrasound. However, approximately 35% of metastatic ALNs do not display suspicious features, which limits the reliability of axillary ultrasonography in accurately assessing ALN status [16].

Because of these challenges, there is a growing need for more efficient, noninvasive methods to predict ALN metastasis. Researchers have developed various imaging-based tools, nomograms, and computer-aided prediction (CAP) systems [17–22]. However, many of these traditional approaches still lack the precision and consistency needed in clinical practice.

In recent years, artificial intelligence, especially deep learning, has made significant advances in medical imaging. CNNs, including well-known models like ResNet and Mask R-CNN, have been used for detecting ALNs from ultrasound and MRI images by learning detailed local features [23]. Radiomics-based methods combined with machine learning models, such as support vector machines and XGBoost, have also been applied to extract handcrafted features from ultrasound, MRI, or PET/CT images to predict ALN status before surgery. While these approaches have shown promising results, CNNs can struggle to capture global spatial relationships, and radiomics pipelines often require extensive preprocessing. These limitations highlight the need for more flexible, data-efficient approaches that can handle limited clinical datasets, such as transformer-based models. To overcome the local feature limitations of CNNs, researchers have adapted transformer architectures [24], which were originally developed for natural language processing, to visual tasks. This adaptation led to the development of Vision Transformers (ViTs) [25], which divide input images into fixed-size nonoverlapping patches and treat them as a sequence of tokens, similar to words in a sentence. Each patch is embedded into a vector and passed through a transformer encoder via self-attention mechanisms. This allows the model to capture both local and global relationships that CNNs might miss. The final image representation is obtained by combining the sequence outputs, typically via mean pooling or a special classification token. ViTs have shown significant potential in medical imaging because they capture global and spatial patterns that CNNs might miss [26].

Despite their advantages, ViTs generally require large amounts of labeled data to perform well, which poses a challenge in clinical applications where carefully labeled ultrasound datasets are often limited. This limitation is particularly important in ALN imaging, where subtle structural patterns and global tissue context play a key role in accurate assessment. To address data scarcity, Data-efficient image transformer (DeiT) was introduced as a more data-efficient variant of ViT. DeiT uses guidance from a stronger model and improved training strategies, allowing it to achieve strong performance even with smaller datasets [27]. In our model, we use DeiT for global feature extraction and combine it with the MLP-Mixer [28] as an attention-free classifier. The MLP-Mixer is simple, reliable, and well suited for modeling spatial and channel interactions, making it an effective choice for ultrasound-based ALN prediction within limited clinical datasets.

In this study, motivated by the above limitations, our main objective was to develop a deep learning model for accurately predicting ALN metastasis in breast cancer patients via grayscale ultrasound images. To achieve this, we propose a hybrid architecture, DeiT-MLP-Mixer, which combines the global feature extraction capabilities of DeiT with a lightweight, attention-free MLP-Mixer-inspired classifier that enhances computational speed and result clarity.

We designed this model to excel in clinical settings, particularly those with limited labeled datasets.

It is trained on a novel, real-world dataset collected from a specialist breast clinic that has not been used in previous studies.

Our contributions can be summarized as follows:

- We propose a new hybrid deep learning model, DeiT–MLP-Mixer, for predicting ALN metastasis from grayscale ultrasound images before surgery. Unlike existing hybrid models that combine CNNs and transformers or rely heavily on attention mechanisms, our approach uses DeiT for global feature extraction and an MLP-Mixer–style classifier for attention-free classification.
- We demonstrate that replacing standard CNN or transformer classification heads with an MLP-Mixer-based classifier improves model stability and generalization, especially when training on small, single-center clinical datasets, which are common in breast ultrasound studies.
- We evaluate the proposed model on a real-world ultrasound dataset of 502 breast cancer patients and show that it consistently performs better than standard CNN and transformer models, highlighting the benefit of separating global feature extraction from task-specific classification.

In summary, our study presents a cutting-edge hybrid model based on the DeiT architecture that combines the best transformer-based deep learning and efficient MLP-style classification. In addition to improving the model’s accuracy and ability to work with limited data, this integration also opens the door for broader use of transformer-driven architectures in medical imaging. By adapting this hybrid approach to grayscale clinical images, our work highlights a promising direction for building practical, noninvasive, and understandable AI tools in real-world healthcare settings.

The rest of this paper is organized as follows: In Related Work, we provide an overview of existing studies relevant to our research. In the Materials and Methods, we describe the proposed DeiT-MLP-Mixer model, including the preprocessing steps and model architecture. We present the experimental setup and evaluate the model’s performance. In the Discussion, we discuss the strengths and limitations of our approach and suggest directions for future research. Finally, in conclusion, we summarize our study and contributions.

### Related works

This section reviews prior studies on breast cancer metastasis detection, with a particular focus on preoperative ALN involvement assessed using ultrasound imaging, which is the primary clinical task addressed in this work. Accurate assessment of ALN involvement plays a critical role in guiding prognosis and treatment strategies for patients with breast cancer. While many studies have focused on primary breast tumor detection [29–32], comparatively fewer works have addressed the equally important problem of preoperative lymph node involvement prediction, which is the main focus of this work.

In recent years, research in this area has increasingly explored how different medical imaging techniques, such as ultrasound can be combined with AI methods to improve diagnostic accuracy, especially in noninvasive preoperative prediction of ALN status. Most of these studies can be broadly categorized into three main groups: CNN-based deep learning approaches, radiomics and machine learning methods, and multimodal fusion strategies. In this section, we review relevant studies on AI-assisted preoperative ALN metastasis prediction, highlighting their methodologies and key findings.

### CNN-based deep learning approaches

Numerous studies have investigated deep learning methods, particularly CNNs, for ALN metastasis detection. In 2017, Bejnordi et al. developed an automated system for detecting lymph node metastases using 399 whole-slide images from two medical centers in the Netherlands, achieving an accuracy of 93.7% [33]. Gutierrez et al. [34] applied CNNs to 118 ultrasound scans from breast cancer patients, with diagnoses confirmed via biopsy, achieving an accuracy of 86.4%. Ren et al. [35] developed a CNN for MRI-based ALN detection using 66 abnormal and 193 normal nodes; the model achieved an accuracy of 84.8%. Lee et al. [36] employed Mask R-CNN for tumor segmentation in 153 ultrasound cases, achieving an accuracy of 81.05% and an AUC of 0.8054. Moon et al. [37] used ultrasound images with tumor-surrounding tissue features for 114 patients, achieving an AUC of 0.8269 and overall accuracy of 81.63%. Butun et al. [38] proposed a ResNet-based framework trained on 220,025 lymph node images from the PCAM dataset, achieving 98.6% accuracy. Chen et al. [39] developed a ResNet18-based CNN using data from 988 women, achieving 84.1% accuracy.

### Radiomics and machine learning approaches

Radiomics-based methods combined with classical machine learning have also been widely explored. Cui et al. [40] applied radiomics to dynamic contrast-enhanced MRI, with SVM achieving an accuracy of 89.54% and AUC of 0.8615. Yang et al. [41] developed a mammography-based radiomic model using SVM, achieving an AUC of 0.85. Song et al. [42] built a PET/CT radiomic model with XGBoost, achieving 80% accuracy and high sensitivity. Li et al. [43] compared traditional and deep learning radiomics for MRI data from 922 patients, with deep learning radiomics performing best (AUC 0.875, 85.2% accuracy). Yao et al. [44] developed an RF-based radiomic model using ultrasound images from 278 patients, achieving an AUC of 0.874.

### Multimodal and integrated approaches

Several studies have explored multimodal fusion to improve ALN prediction performance. Guo et al. [45] combined ultrasound and advanced feature analysis, achieving 95.2% accuracy. Zheng et al. [46] used a 3D CNN with ultrasound and shear wave elastography, achieving an AUC of 0.902 and outperforming conventional diagnostics. Tang et al. [47] integrated ultrasound and MR images using a dual-branch CNN for 479 patients, achieving an AUC of 0.92. Cai et al. [48] combined ultrasonographic parameters with breast-specific gamma imaging in 334 patients, achieving an accuracy of 73.7% and an AUC of 0.794.

In the above section, we discussed some related works that used deep learning [33–39], radiomics and machine learning techniques [40–44], and multimodal fusion strategies [45–48], with their main characteristics and findings summarized in Table 1.

While these methods have shown encouraging results, several challenges remain. CNN-based models primarily focus on local features, which can limit their ability to capture the overall structure of ultrasound images. Radiomics pipelines rely on handcrafted features and often require extensive preprocessing. Multimodal approaches, while effective, depend on additional imaging modalities that may not be routinely available in preoperative clinical workflows. Many deep learning models, especially CNN-based or pretrained architectures, also rely on large, carefully labeled datasets that are difficult to collect in routine practice. In addition, repeated convolution operations can reduce spatial resolution, particularly when applied to small or lower-quality ultrasound images.

Despite the increasing interest in transformer-based models for medical image analysis, most current approaches for ALN metastasis prediction still rely on conventional CNNs or standard ViTs. Although ViTs are effective at modeling global relationships, they typically require large-scale training data and may not generalize well to limited clinical ultrasound datasets. DeiT address this issue through knowledge distillation; however, existing DeiT-based methods commonly use simple linear or attention-based classification heads, which do not fully leverage interactions between image patches.

To overcome these limitations, we propose a hybrid DeiT-MLP-Mixer model that combines data-efficient global feature extraction with an MLP-Mixer–inspired classifier that explicitly models spatial and channel-level interactions between patches. This design enables effective learning from relatively small grayscale ultrasound datasets while remaining computationally efficient and interpretable. By directly addressing the limitations of existing transformer-based approaches, the proposed method offers a novel and practical contribution to AI-assisted ultrasound analysis for preoperative ALN metastasis prediction.

**Table 1:** Summary of previous work on ALN prediction

Model	Architecture	Dataset	Data Type	Results	Limitations
Bejnordi et al., 2017 [33]	CNN	399 whole-slide images	Histopathology	Accuracy 93.70%	Small, 2-center dataset, not ultrasound-based
Gutierrez et al., 2020 [34]	CNN	118 images	Ultrasound	Accuracy 86.40%	Small, single-center ultrasound dataset
Ren et al., 2020 [35]	CNN	259 MRI nodes (66 abnormal, 193 normal)	MRI	Accuracy 84.80%	Small dataset, MRI only
Lee et al., 2020 [36]	Mask R-CNN	153 patients	Ultrasound	Accuracy 81.05%	Moderate dataset, segmentation-dependent
Moon et al., 2021 [37]	CNN	114 patients	Ultrasound	Accuracy 81.63%	Small, single-center ultrasound dataset
Butun et al., 2021 [38]	ResNet-based CNN	PCAM dataset (220,025 images)	Histopathology	Accuracy 98.60%	Not breast-specific, large curated dataset
Cui et al., 2019 [39]	Radiomics + SVM	121 patients (DCE-MRI)	MRI	Accuracy 89.54%	MRI only, handcrafted features
Yang et al., 2020 [40]	Radiomics + SVM	147 patients	Mammography	AUC 0.85	Small, single-center dataset, manual feature extraction
Song et al., 2020 [41]	Radiomics + XGBoost	100 patients	PET/CT	Accuracy 80%	Small, single-center dataset, PET/CT modality only
Chen et al., 2020 [42]	ResNet18 CNN	988 patients	Ultrasound	Accuracy 84.1%	Moderate dataset, single-center ultrasound dataset
Li et al., 2021 [43]	Deep learning radiomics	922 patients	MRI	Accuracy 85.2%	MRI only, comparison of handcrafted vs. deep learning features
Guo et al., 2021 [44]	Ultrasound + advanced feature analysis	Ultrasound	Ultrasound	Accuracy 95.2%	Requires feature engineering; single-modality ultrasound
Zheng et al., 2021 [45]	3D CNN	Ultrasound + shear wave elastography	Ultrasound	AUC 0.902	Small, single-center dataset; multimodal US only
Tang et al., 2021 [46]	Dual-branch CNN	479 patients	US + MRI	AUC 0.92	Multimodal dataset, may not be routinely available clinically
Yao et al., 2021 [47]	RF-based radiomics	278 patients	Ultrasound	AUC 0.874	Small, single-center ultrasound dataset
Cai et al., 2021 [48]	Ultrasound + gamma imaging	334 patients	Ultrasound + Gamma Imaging	Accuracy 73.70%	Multimodal, not routine in preoperative workflow

## Materials & methods

In this study, we present a deep learning model called DeiT-MLP-Mixer for classifying ALN status in breast cancer patients via grayscale ultrasound images. The model combines two main components, a DeiT for feature extraction and an MLP-Mixer-inspired classification layer. The DeiT module breaks each image into small patches and learns how these parts relate to each other, helping the model capture the most important global patterns across the entire image. This is followed by a simple classification layer that integrates information from all parts of the image to make a final prediction. Unlike many traditional models, our approach avoids the use of attention mechanisms in the last stage, which helps reduce complexity and speed up processing. By focusing on both local and global features, the model is designed to perform well even in real-world clinical settings where data are limited.

### Data-efficient vision transformers (DeiT)

DeiT, introduced by Touvron et al. (2021) [27], is an efficient version of the ViT [25] designed to perform well even with limited training data, which is common in medical imaging tasks. Like ViT, DeiT splits each image into small patches and changes them into tokens via a simple linear layer. These tokens go through several transformer blocks that learn the spatial relationships between different regions of the image. Standard DeiT models use a classification (CLS) token, which gathers the key information needed for the final prediction. A special version, called distilled DeiT, also adds a distillation (DIST) token. This token is trained through knowledge distillation, where it learns from a stronger pretrained CNN “teacher” model. During training, both the CLS and DIST tokens work together to improve learning. At the prediction stage, the model averages its outputs to make the final decision.

**Fig. 1:**
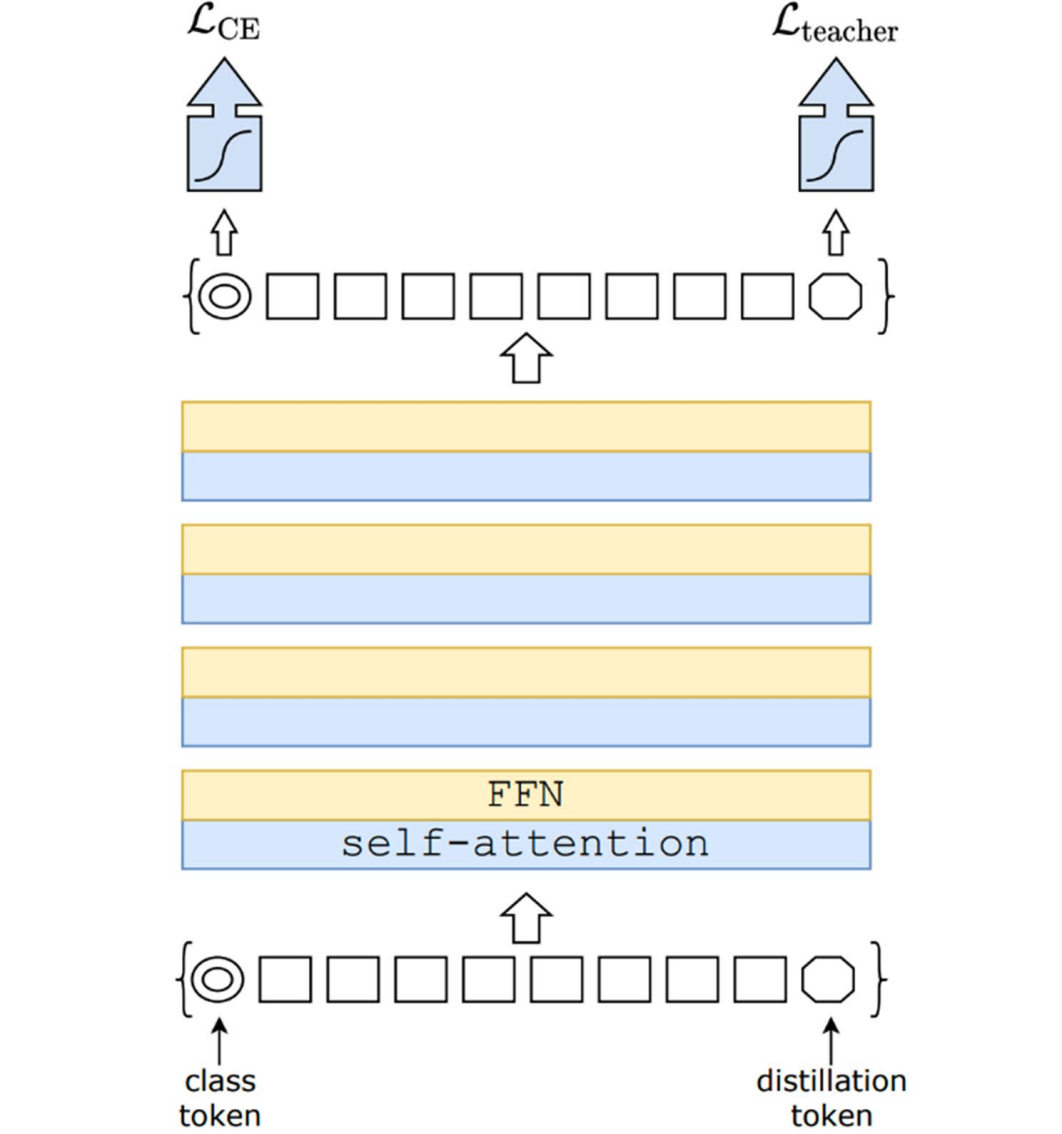
Architecture of the DeiT model [25]

DeiT offers several versions designed for different computational needs: DeiT-Tiny (5 M parameters, 192 × 192 input), DeiT-Small (22 M parameters), DeiT-Base (86 M parameters, both with 224 × 224 input), and DeiT-Base-384 (86 M parameters, 384 × 384 input for higher resolution). There are also distilled versions, such as DeiT-Base-Distilled, which use a DIST token to help the model learn better. Figure 1 shows the architecture of the DeiT model. In this study, we use the DeiT-Base-Distilled model with 224 × 224 inputs as part of our DeiT-MLP-Mixer. This setup allows DeiT to use the strengths of the teacher model while remaining efficient and easier to train on smaller datasets such as the one we use here.

### MLP-mixer

**Fig. 2:**
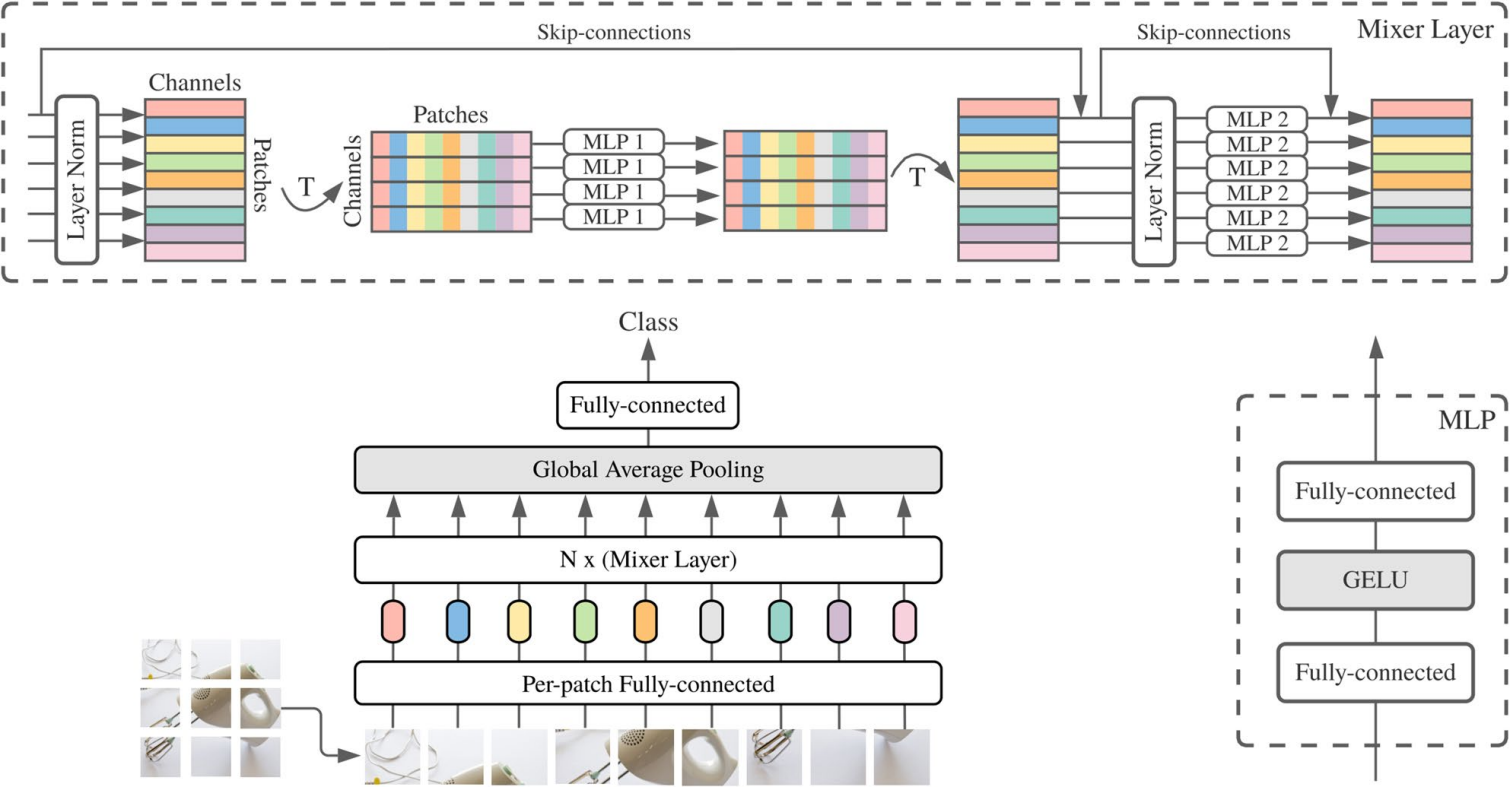
Architecture of the MLP-mixer [42]

Our DeiT-MLP-Mixer model includes a classifier inspired by the MLP-Mixer architecture, which is designed to handle image features in an efficient and easy-to-understand way. In the work of Tolstikhin et al. [28], this classifier uses multilayer perceptrons (MLPs) to perform two key operations, mixing information across the spatial dimensions of the image and mixing information across different feature channels. These steps work together to combine and refine the extracted features. Unlike traditional transformers, this approach does not rely on attention mechanisms, making it lighter to run and more practical for clinical use. The architecture of the MLP-Mixer is displayed in Fig. 2. The following section explains how spatial and channel mixing work together to improve feature processing, leading to accurate classification of lymph nodes.

### Spatial mixing

Spatial mixing combines information from several parts of the medical image, capturing global interactions between image patches such as lymph nodes and surrounding tissues. By combining information from all patches, the model can understand the broader spatial context of the axillary region, which is crucial for detecting lymph node involvement patterns.

### Channel mixing

Channel mixing improves the feature representation within each image patch, enhancing local details such as texture or intensity gradients that distinguish involved from noninvolved lymph nodes. This process focuses on improving the features of individual patches, ensuring that subtle pathological patterns are captured effectively. By highlighting these local properties, channel mixing increases the model’s sensitivity to fine-grained diagnostic cues, which is essential for accurate classification in medical imaging.

### Dataset

This study included ultrasound data from 502 breast cancer patients collected from the electronic medical records of a specialist breast clinic in Iran. There was no identifying or personal information in the collection; it only contained ultrasound images. The use of these images for study was approved by the University of Isfahan Ethics Committee under protocol number [IR.MUI.NUREMA.REC.1404.075].

**Fig. 3:**
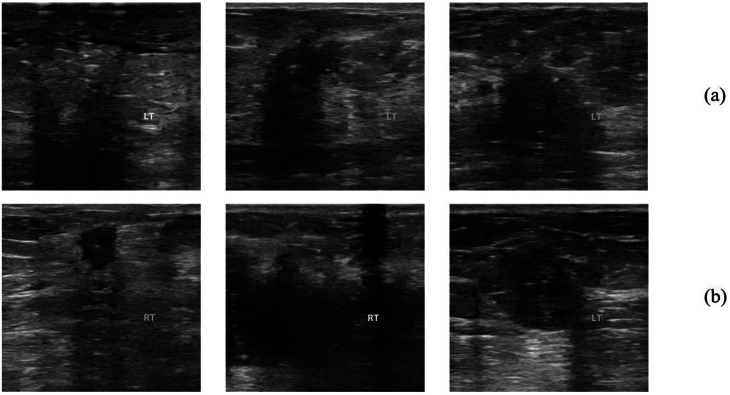
(**a**) Involved and (**b**) noninvolved axillary lymph node ultrasound images from the dataset

All ultrasound scans were performed at the clinic by an experienced breast specialist via a Mindray Z5 Diagnostic Ultrasound System equipped with a compatible 75L38EA linear-array probe operating between 5.0 and 7.5 MHz. All images were obtained using a high-frequency linear probe with a vertical probe orientation. Image acquisition was performed on a single ultrasound system from the same manufacturer and software version, which minimized variability related to device-specific image quality and acquisition settings. While this ensured consistency across the dataset, it may limit generalizability to images acquired using different ultrasound systems, which is acknowledged as a limitation and discussed further in the Discussion section. One grayscale ultrasound image was obtained from each patient before surgery, and the lymph node status was determined from postoperative pathology reports. All cases without lymph node involvement were confirmed by sentinel lymph node biopsy. Cases classified as lymph node–positive included only those with micro-metastasis, which have a clear impact on treatment planning. All lymph node–positive cases were confirmed by histopathological examination. All cases included in this study involved single tumors, multicentric and multifocal tumors were excluded from the analysis. These findings revealed that 283 patients had noninvolved ALNs, and 219 had involved nodes. Examples of both involved and noninvolved ultrasound images are randomly shown in Fig. 3. The images were preprocessed and labeled accordingly for binary classification of lymph node involvement.

### Data augmentation and transformation

The original dataset consisted of 502 ultrasound images, with one image per patient. These images were first split on a patient basis to prevent any data leakage between training, validation, and testing. Given the moderate class imbalance in the dataset (283 noninvolved vs. 219 involved cases), we used stratified sampling when splitting the data into training, validation, and test sets to maintain consistent class distribution across all subsets, rather than artificially rebalancing the data. No additional class-balancing methods, such as class weighting or resampling, were applied during training. To ensure a careful and unbiased evaluation, we followed a strict split → augment →evaluate workflow. As summarized in Table 2, the data were divided into 70% for training, 10% for validation, and 20% for testing, and were then expanded through offline augmentation to a total of 1,506 images, with 1,053 used for training, 151 for validation, and 302 for testing.

During model training and evaluation, different image processing pipelines were used through the data loaders. For all images, the first step was standardization, which involved converting the single-channel grayscale images to three-channel RGB and resizing them to a fixed size of 224 × 224 pixels. This provided a consistent input format and ensured compatibility with the DeiT-base architecture.

For training, a stochastic pipeline was applied after standardization. In this step, images were augmented on the fly to represent common variations observed in real clinical ultrasound images. For training, images were augmented on the fly to improve generalizability. This included random resized cropping to 224 × 224 pixels, horizontal flips (probability = 0.5), random rotations (± 15°), brightness and contrast adjustments (± 0.2), random affine transformations (± 10°), and color jitter to randomly perturb the brightness and contrast. Because the original ultrasound images were grayscale, RGB conversion was performed by duplicating the single grayscale channel across three channels to match the input requirements of the DeiT-based architecture. In contrast, the validation and test sets were processed using a fixed and deterministic pipeline, consisting only of RGB conversion and resizing to 224 × 224 pixels, without any random transformations. All images were normalized using a mean and standard deviation of 0.5 per channel to ensure compatibility with the DeiT-base architecture.

All scans were collected by experienced breast specialists following a standardized protocol. This ensured consistent probe pressure, angle, and gain settings, focusing specifically on the relevant axillary region. In addition, geometric augmentation during training was used to simulate slight changes in probe angle and patient positioning, helping the model handle the normal variations in anatomy and positioning seen in clinical practice. Although data augmentation helped increase the size of the training set, it only adds artificial variations based on the existing images and cannot fully represent the diversity found in independent, multi-institutional datasets. Therefore, augmentation cannot replace external validation, and this limitation is discussed further in the Discussion section.

**Table 2:** Involved and noninvolved axillary lymph node ultrasound images from the dataset

Class	Training	Validation	Test	Total
Involved	459	66	132	657
Non-Involved	594	85	170	849
Total	1053	151	302	1506

### Proposed DeiT-MLP-mixer architecture

The proposed DeiT-MLP-Mixer model consists of two main components: a DeiT-based feature extractor and an MLP-Mixer-inspired classifier. The feature extraction stage is built upon the data-efficient image transformer (DeiT-base-distilled-patch16–224). Each input image (224 × 224 pixels, three channels) is divided into 196 nonoverlapping patches of 16 × 16 pixels. These patches are flattened and projected into 768-dimensional embedding vectors, forming a sequence of 196 patch embeddings. Two additional tokens, the CLS token and the DIST token, are appended, resulting in a total of 198 tokens. Learnable positional embeddings are added to preserve spatial information, and the sequence is passed through 12 transformer layers. Each layer consists of multihead self-attention and feedforward sublayers with residual connections and layer normalization. Standard DeiT architectures use the CLS and DIST tokens to compress global information into a single vector for classification. While useful for summarizing the image, these tokens lose the detailed spatial layout of the feature map. The MLP-Mixer, on the other hand, works with a fixed sequence of image patches to learn relationships between specific anatomical regions, such as the boundary of a lymph node compared to the surrounding tissue. Including the CLS or DIST tokens would disrupt this spatial structure. That’s why we discard these summary tokens and let the MLP-Mixer focus directly on the 196 patch embeddings, using only the raw spatial features from each patch. The output tensor at this stage has the shape (batch_size, 196, 768), which represents the features extracted from each image patch. The classification stage replaces traditional attention-based or linear heads with an MLP-Mixer-style classifier with a full two-layer MLP-Mixer stack *(L = 2).* Each of the two layers sequentially performs spatial and channel mixing within a post-normalization residual structure. In the spatial mixing step, the tensor is transposed to (batch_size, 768, 196), allowing the model to capture relationships across spatial patches. A linear layer maps the 196 patch dimension to a hidden size of 512, followed by a GELU activation function and dropout at a rate of 0.3 to reduce overfitting. A second linear layer then maps the hidden representation back to 196 dimensions, followed by another dropout layer. The result is transposed back to (batch_size, 196, 768) and added to the original input via a residual connection. This stage enables the model to learn contextual dependencies between different regions of the image, such as lymph node structures and surrounding tissue. Next, the channel mixing stage operates on each individual patch to integrate information across the 768 feature channels. The process mirrors spatial mixing: a linear layer expands the channel dimension to 512, followed by GELU activation and dropout, then projects back to 768 features with an additional dropout and residual connection. This enhances the feature representation by emphasizing interchannel relationships, which is important for recognizing subtle variations in medical imaging. After the completion of both mixing layers, layer normalization is applied to the tensor to stabilize training and improve generalization. The tensor is then transposed to (batch_size, 768, 196), and adaptive average pooling is used to reduce the patch dimension, producing a final tensor of shape (batch_size, 768). This compact feature vector is passed through a final linear layer, which outputs two logits corresponding to the binary classification of lymph node status (involved vs. noninvolved). During inference, a Softmax function is applied to convert logits into class probabilities.

**Fig. 4:**
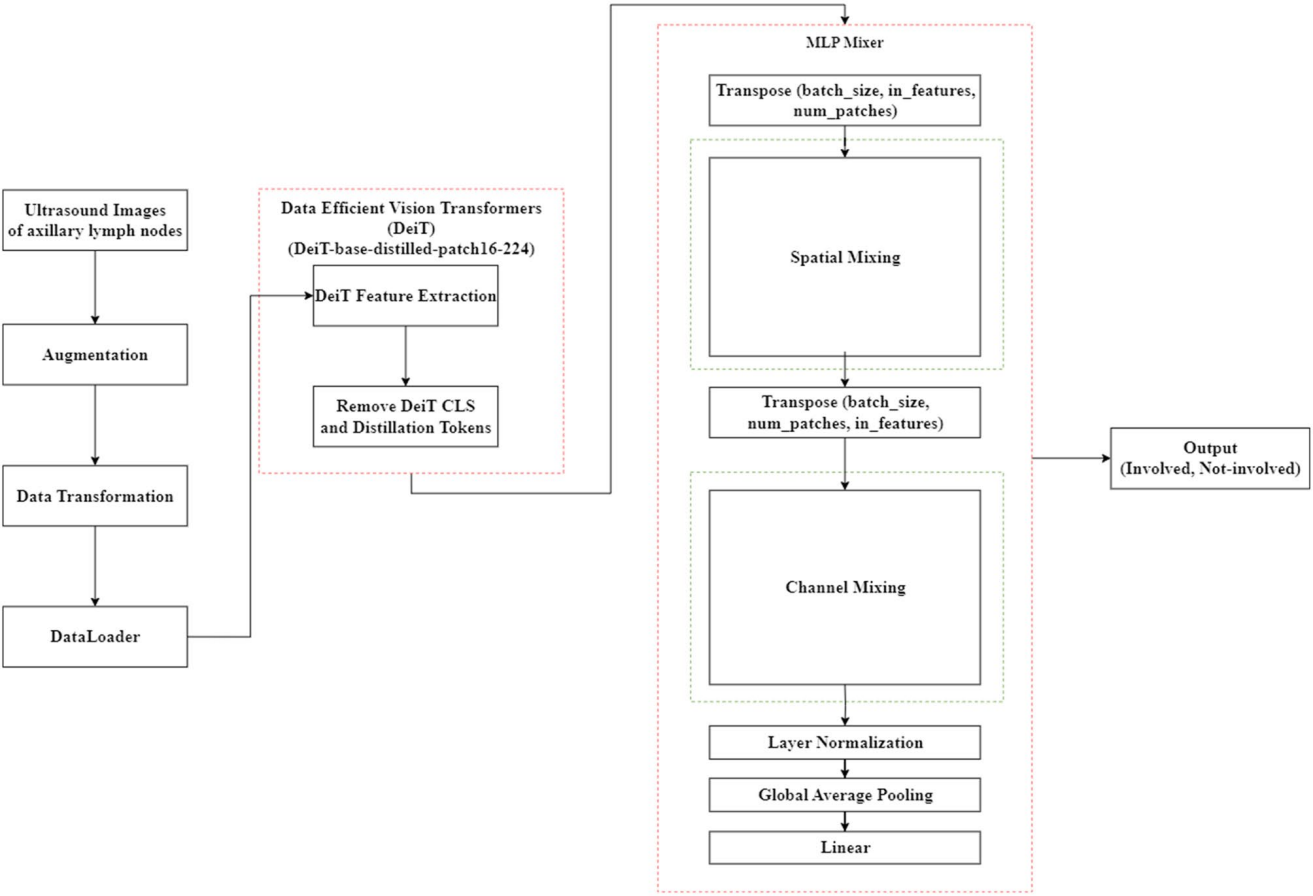
Flowchart of the proposed DeiT-MLP-mixer model

The flowchart shown in Fig. 4 shows the overall process of our proposed method for classifying ALN status. It starts with a set of 224 × 224 ultrasound images that go through a series of transformations, including converting them to RGB format and normalizing them to prepare for model input. These transformed images are then organized into batches via DataLoader. The next step involves extracting meaningful features from the images via DeiT, which divides each image into small patches and learns how these parts relate to each other. Since the class and DIST tokens are not needed in our setup, they are removed, and the remaining features are then passed into the MLP-Mixer, which processes them through spatial and channel mixing layers. Finally, the model outputs a binary classification, identifying each image as either involved or not involved on the basis of lymph node status.

**Fig. 5:**
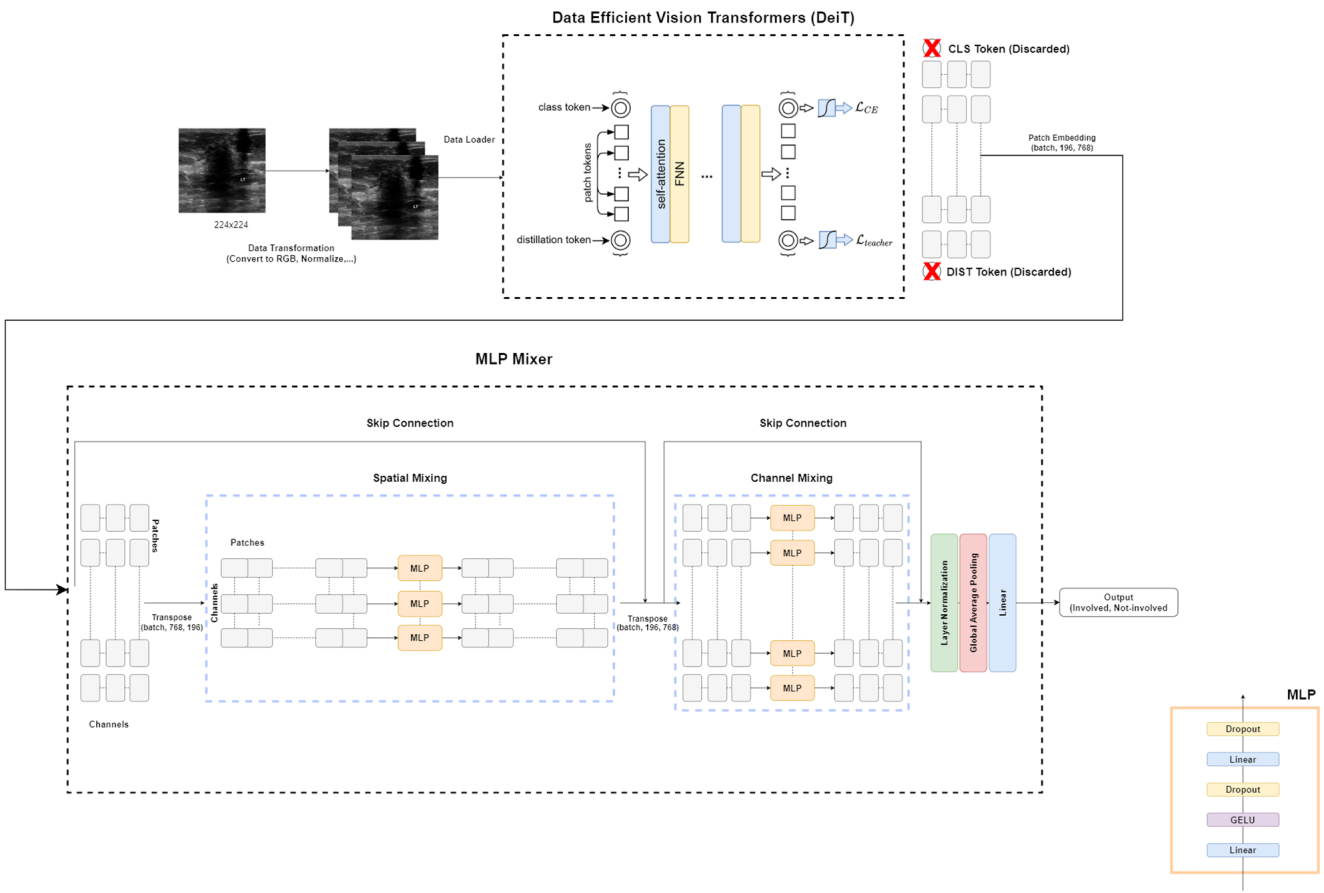
Architecture of the proposed DeiT-MLP-Mixer model

The primary goal of the proposed architecture, which is shown in Fig. 5, is to combine two important components, one that focuses on capturing the overall patterns across the entire ultrasound image and the other that integrates the most important details to make the final prediction. This combination allows the model to work faster and more efficiently without losing accuracy. It is especially useful in settings such as hospitals or clinics, where data availability is limited and quick, reliable results are required for patient care.

## Results

The experiments were conducted in a Python environment via Kaggle notebooks. The system was equipped with an NVIDIA Tesla T4 GPU (16 GB VRAM), 32 GB of RAM, and an Intel(R) Xeon(R) CPU @ 2.00 GHz. Model development and training were performed via the PyTorch framework (version 2.5.1), along with the transformer library from Hugging Face (version 4.47.0), to access the pretrained DeiT-base-distilled-patch16-224 model. Additional tools included NumPy (1.26.4) for numerical operations, scikit-learn (1.2.2) for performance metrics (accuracy, precision, recall, and F1 score), Matplotlib (3.7.5) for visualization, and torchvision (0.20.1) for image transformations and dataset handling.

Hyperparameters were selected based on prior literature on transformer-based medical imaging models and preliminary pilot experiments. The DeiT-Mixer model was trained using the AdamW optimizer with an initial learning rate of 2 × 10⁻⁵, following best practices for fine-tuning large pretrained transformers to preserve learned representations and ensure stable convergence on limited datasets. A weight decay of 0.01 was applied to help mitigate overfitting, and a batch size of 32 was chosen to balance GPU memory constraints, computational efficiency, and gradient stability. With 1,053 training images, this batch size resulted in 33 batches per epoch. After augmentation, the final training dataset consisted of 1,506 grayscale ultrasound images. Training was performed for up to 50 epochs, with early stopping based on validation accuracy and a patience of five epochs to prevent overfitting and avoid unnecessary computation. Binary cross-entropy loss was used for the binary classification of involved versus noninvolved lymph nodes, which is appropriate for distinguishing these two classes. To further improve convergence, a ReduceLROnPlateau scheduler was employed, reducing the learning rate by a factor of 0.1 if the validation loss did not improve for five consecutive epochs. The complete set of training parameters is summarized in Table 3.

On the NVIDIA Tesla T4 GPU, the average training time per epoch was approximately 40 s. Early stopping was triggered at epoch 44, resulting in a total training time of approximately 29 min.

**Table 3:** Training parameters for the proposed model

Parameter	Value
Batch size	32
Epochs	50
Optimizer	AdamW
Learning rate	2 × 10⁻⁵
Weight decay	0.01
Loss	Cross-entropy
Patience	5

The model’s performance was evaluated via common assessment metrics, including accuracy, precision, recall, specificity, and the F1 score [49]. Each provides a different perspective on how effectively the model distinguishes between involved and noninvolved lymph nodes. These metrics are defined via the following terms: FN stands for false negatives, TN for true negatives, TP for true positives, and FP for false positives.

Accuracy: Accuracy in classification is a metric that measures the proportion of correctly classified instances out of all cases in the dataset. It is one of the most commonly used metrics for evaluating classification performance and is calculated via the following formula:

1 $$Accuracy=\frac{TP+TN}{TP+TN+FP+FN}$$

Precision: Precision in classification is a metric that measures the accuracy of positive predictions made by a classifier. It represents the ratio of true positive predictions to the total number of positive predictions made by the classifier, regardless of the actual class of the instances. The formula is as follows:

2 $$Precision=\frac{TP}{TP+FP}$$

Recall: Recall, also known as the sensitivity or true positive rate, is a classification metric that reflects a classifier’s ability to identify all relevant instances accurately or the proportion of true positive examples correctly detected. It is computed via the following formula:

3 $$Recall=\frac{TP}{TP+FN}$$

Specificity: Specificity in classification, also known as the true negative rate, reflects a classifier’s ability to identify all negative cases accurately. This metric measures the proportion of real negative instances correctly detected by the classifier out of all actual negative instances. Specificity is calculated via the following formula:

4 $$Specificity=\frac{TN}{TN+FP}$$

F1 score: The F1 score combines precision and recall into a single value, achieving a balance between the two measures. It is calculated via the harmonic mean of precision and recall and assigns equal weights to both measures. The formula for computing the F1 score is as follows:

5 $$F1-Score=2\times\frac{Precision \times Recall}{Precision+Recall}$$

Confusion matrix: A confusion matrix shows a classification model’s performance by showing the number of true positive (TP), true negative (TN), false positive (FP), and false negative (FN) predictions on a dataset. The matrix is square, with rows representing actual classes and columns representing predicted classes. The main diagonal of the matrix displays the number of correct predictions (TP and TN), whereas the off-diagonal elements represent incorrect predictions (FP and FN). The confusion matrix evaluates classification performance by measuring accuracy, precision, recall, and other measures.

Loss function: The loss function is an important component of machine learning models that assesses the difference between expected and actual values to determine how well the model performs during training. It is essential to focus the optimization process toward reducing this difference and increasing the model’s accuracy. In this study, we used the cross-entropy loss function. Cross-entropy loss, commonly known as log loss, is a popular loss function in classification tasks, especially in settings with several classes. It measures the difference between the predicted probability distribution and the actual distribution of class labels. Mathematically, for a classification problem with *N* samples and *K* classes, where *y*_*ik*_ denotes the true label (1 if sample *i* belongs to class *k*, 0 otherwise) and *p*_*ik*_ denotes the predicted probability of sample *i* belonging to class *k*, the cross-entropy loss is given by:

6 $$Cross\mathrm{-}EntropyLoss=-\frac{1}{N}\sum_{i=1}^{N}\sum_{k=1}^{K}{y}_{ik}\mathrm{log}\left({p}_{ik}\right)$$

The loss penalizes false predictions more heavily, particularly when the predicted probability differs greatly from the true label. This property helps the model assign high probabilities to the correct class labels.

To better understand how the model learned during training, we monitored the accuracy and loss over each epoch. These plots provide a clear picture of how the model’s performance evolves over time and help us identify potential issues such as underfitting, overfitting, or instability in the learning process.

Accuracy per epoch graph: The accuracy per epoch graph shows a machine learning model’s performance on training and validation data over multiple epochs. Each epoch represents a complete trip through the whole training dataset. The accuracy per epoch graph shows the number of epochs on the *x*-axis and the accuracy on the *y*-axis. The accuracy is shown by two lines or curves: one for training data and one for validation data. The monitoring accuracy per epoch is used to evaluate the model’s ability to learn from training data and apply it to new data.

Loss per epoch graph: The loss per epoch graph shows a machine learning model’s error or cost in training and validation data over multiple epochs. The loss per epoch graph usually displays the number of epochs on the x-axis and the loss value on the y-axis. Similar to the accuracy per epoch graph, it shows two lines or curves: one for the loss of training data and the other for the loss of validation data. The loss per epoch graph shows the model’s convergence and performance during training, with lower values suggesting a better fit.

### Experimental results

We evaluated the performance of the proposed DeiT-MLP-Mixer model and compared it with a wide range of state-of-the-art architectures, including the standard ViT, DeiT, and several widely used pretrained CNNs such as ConvNext, EfficientNet, DenseNet, InceptionV3, MobileNetV2, and VGG-16. To ensure a fair comparison, all models were trained and tested on the same dataset under identical experimental conditions. In addition, we performed an internal ablation study within the DeiT framework, comparing our proposed spatial-mixing head against three standard configurations: CLS+DIST (standard distilled DeiT), CLS-only, and Patch Pooling (Global Average Pooling).

As summarized in Table 4, we report accuracy, precision, recall, specificity, F1 score, and loss for each model, along with 95% confidence intervals for key metrics. The DeiT-MLP-Mixer achieved the best overall performance, with an accuracy of 95.70%, precision of 0.9760, recall of 0.9242, specificity of 0.9867, F1 score of 0.9494, and a test loss of 0.1345. These results show that integrating the MLP-Mixer head for explicit spatial patch interaction improves diagnostic reliability, particularly in identifying non-involved lymph nodes, compared with both pretrained CNNs and standard transformer approaches that rely on summary tokens.

**Fig. 6:**
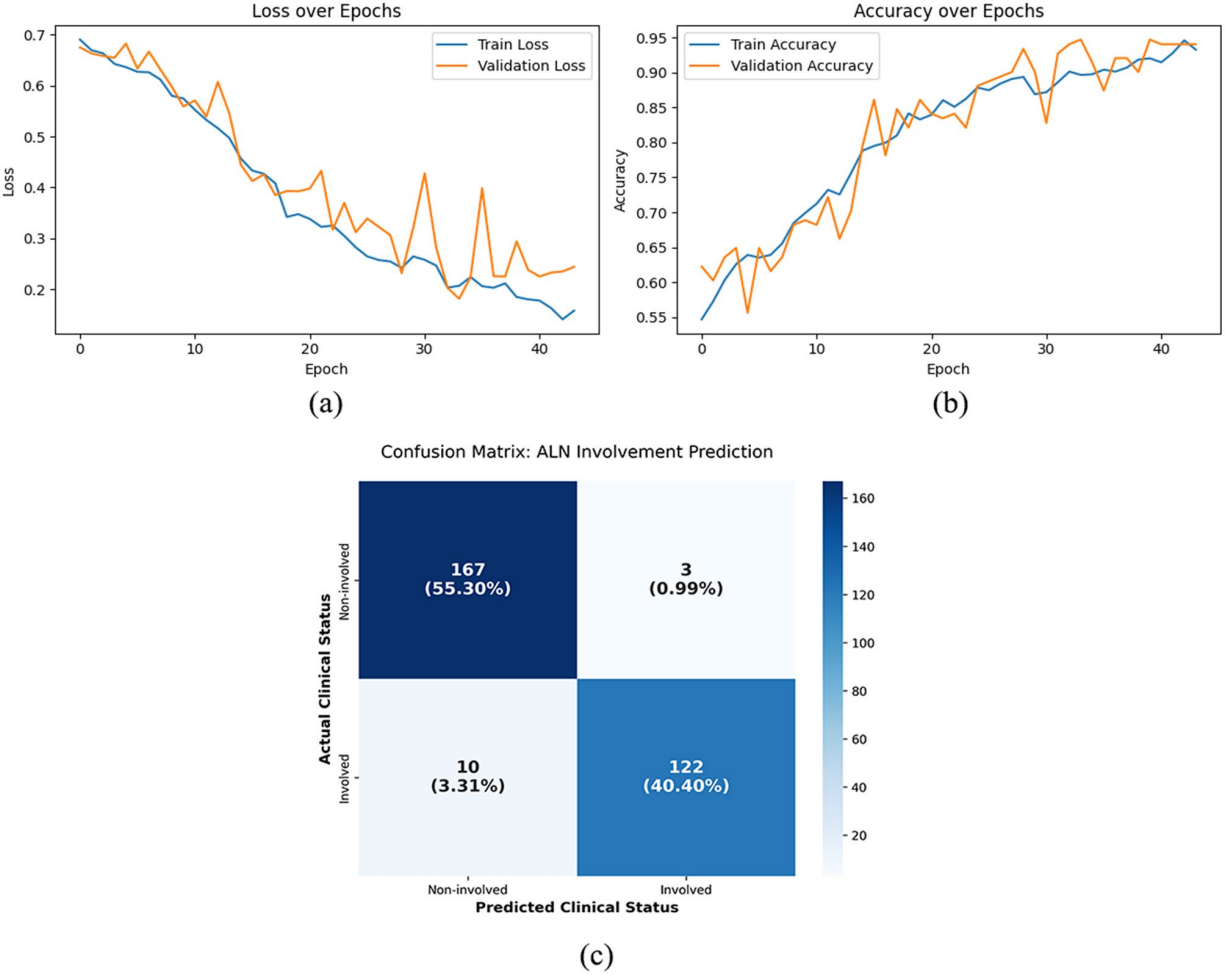
(**a**) Loss per epoch graph, (**b**) accuracy per epoch graph, (**c**) confusion matrix of the proposed model

The training and validation performance of the DeiT-Mixer model over 44 epochs (with early stopping triggered) is illustrated in Fig. 6. The accuracy graph shows a strong increasing trend from 54.70% in the first epoch to 93.26% by epoch 44, with the validation accuracy reaching 94.70% in epoch 40. This suggests consistent learning and little evidence of overfitting. The loss curve also shows a continuous decrease in training loss (from 0.6907 to 0.1580) and validation loss (reaching 0.1814 by epoch 34), reflecting good generalizability to previously unseen data. The confusion matrix confirms the model’s reliability, with high recall and precision scores. This means that the model can accurately detect involved lymph nodes while minimizing false positives and negatives, an essential quality for making confident clinical decisions before surgery.

**Table 4:** Different performance measures for the experimental models on the validation data

Model	Accuracy (%) [95% CI]	Precision	Recall [95% CI]	Specificity [95% CI]	F1 Score	Loss
DeiT (CLS + DIST)	94.37 [91.8–97]	0.9318	0.9305 [88.7–97.4]	0.9176 [87.6–95.9]	0.9306	0.1946
DeiT (CLS Only)	94.37 [91.8–97]	0.9078	0.9697 [94.0%-99.9%]	0.9235 [88.4%-96.3%]	0.9377	0.1404
DeiT (Patch Pooling)	93.71 [91.0%-96.%]	0.9248	0.9318 [88.9%-97.5%]	0.9412 [90.6%-97.7%]	0.9283	0.1888
ViT	91.39 [88.2–94.6]	0.9202	0.9139 [86.6–96.2]	0.9235 [88.4–96.3]	0.9128	0.2452
InceptionV3	82.12 [77.8–86.4]	0.8421	0.7273 [65.1–80.3]	0.8941 [84.8–94]	0.7805	0.4466
MobileNetV2	81.13 [76.7–85.5]	0.8378	0.7045 62.7–78.2]	0.8941 [84.8–94]	0.7654	0.4092
VGG16	80.13 [75.6–84.6]	0.8750	0.6364 [55.4–71.8]	0.9294 [89.1–96.8]	0.7368	0.4538
ConvNext	92.38 [89.4–95.4]	0.8921	0.9394 [89.9–98]	0.9118 [86.9–95.4]	0.9151	0.2469
EfficientNet	89.74 [86.3–93.2]	0.9391	0.8182 [75.2–88.4]	0.9588 [92.9–98.9]	0.8745	0.2214
DenseNet	91.39 [88.2–94.6]	0.8897	0.9167 [87-96.4]	0.9118 [86.9–95.4]	0.9030	0.2350
**DeiT-MLP-Mixer**	**95.70 [93.4–98]**	**0.9760**	**0.9242 [87.9–96.9]**	**0.9867 [96.9–100]**	**0.9494**	**0.1345**

To evaluate the clinical feasibility of the proposed DeiT-MLP-Mixer, we measured its computational requirements. As shown in Table 5, the model has 88.22 million parameters and requires 17.57 billion FLOPs per forward pass. Tests on an NVIDIA Tesla T4 GPU showed an average processing time of 14.85 milliseconds per image. This corresponds to a throughput of about 67 images per second. This latency is comparable to conventional CNN-based models evaluated in this study and supports its suitability for real-time or near–real-time clinical workflows. Table 5 also includes the same efficiency metrics for all baseline models, allowing for a clear comparison of performance relative to computational cost.

**Table 5:** Comprehensive comparison of performance and computational complexity

Model	Params (M)	FLOPs (G)	Latency (ms)
VGG16	138.36	15.47	9.67
ConvNext	88.57	15.37	14.89
DenseNet121	7.98	2.90	15.28
MobileNetV2	3.5	0.33	5.43
EfficientNet-B0	5.29	0.42	8.48
InceptionV3	23.83	5.75	12.18
ViT	86.42	16.86	15.49
DeiT (Distilled)	87.18	16.95	15.76
**Proposed Model (DeiT-MLP-Mixer)**	**88.22**	**17.57**	**14.85**

**Fig. 7:**
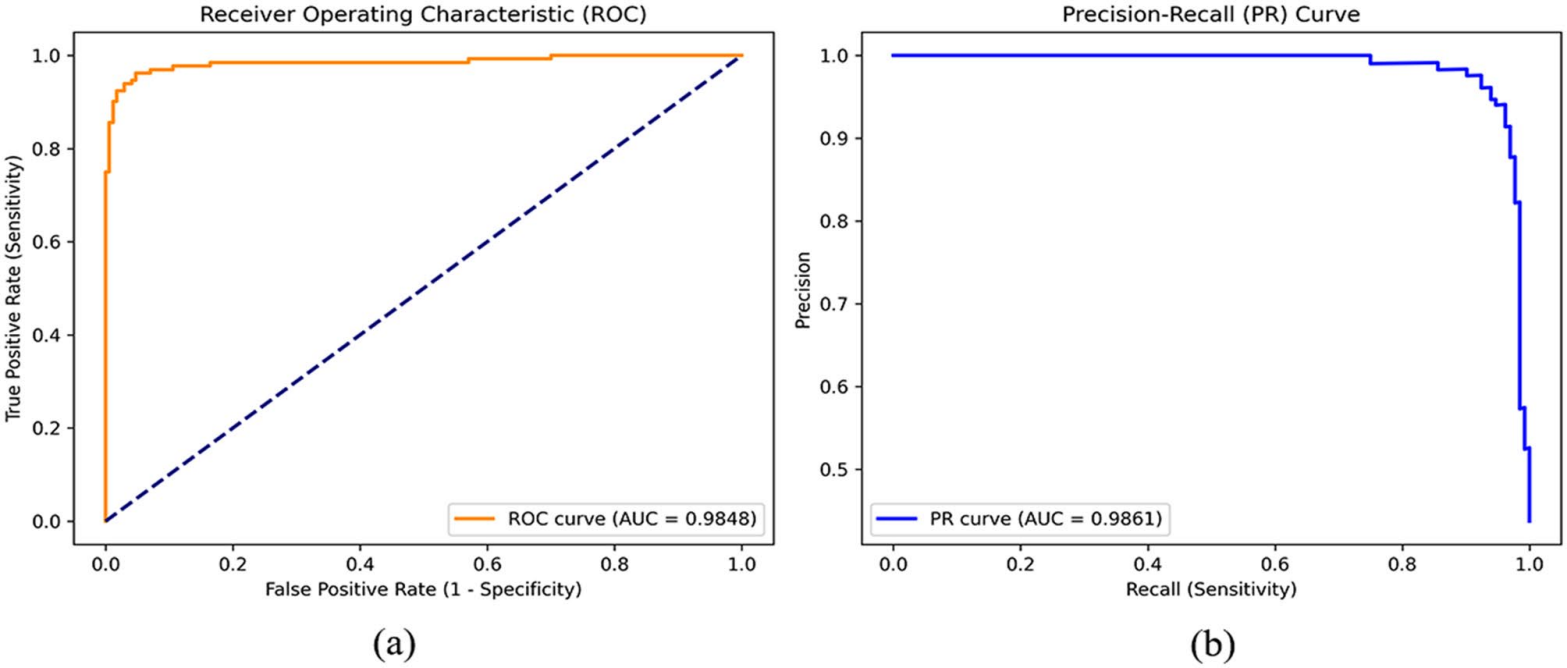
ROC and PR curves for the DeiT-MLP-Mixer

To further assess the diagnostic reliability and clinical usefulness of the DeiT-MLP-Mixer, we conducted a comprehensive performance analysis using Receiver Operating Characteristic (ROC) and Precision–Recall (PR) curves, as shown in Fig. 7. The model demonstrated excellent discriminative ability, achieving a ROC curve with an AUC of 0.9848 and a precision–recall curve with an AUC of 0.9861, indicating strong performance in distinguishing between involved and non-involved lymph nodes across a wide range of decision thresholds.

We also evaluated the reliability of the model’s probability estimates using the Brier score. The low Brier score of 0.0378 indicates good calibration, meaning that the predicted risk scores closely reflect the true clinical outcomes. This is particularly important for preoperative decision-making. For example, when the model was operated at a high-sensitivity threshold of 95.0% to minimize missed metastases, it still maintained a high specificity of 95.29%.

Table 6 provides a comparison of the proposed model with various approaches discussed in related works, offering a broader perspective on their approach, dataset used, and results gained.

**Table 6:** Comparison of the proposed model with other models in related works

Model	Architecture	Dataset	Data Type	Accuracy (%)
Deep learning for metastasis [29]	CNN	399 whole-slide images	Histopathology images	93.70
Deep learning with CNN [30]	CNN	118 images	Ultrasound	86.40
Deep learning [32]	Mask R-CNN	153 patients	Ultrasound	81.05
DL + Transfer learning [34]	ResNet + 1Cycle policy	PCAM dataset (220,025 images)	Histopathology	98.60
Radiomics + ML [35]	SVM	121 patients (DCE-MRI)	MRI	89.54
Hybrid DL Radiomics [39]	CNN + traditional features	922 patients	MRI	85.20
Deep Learning Radiomics [40]	CNN	937 patients	Ultrasound	95.20
Multimodal DL [42]	Dual-branch CNN	479 patients	Ultrasound + MRI	92.00
ML Radiomics [43]	RF (best among 4 classifiers)	278 patients	Ultrasound	87.40
ML + multimodal imaging [44]	SVM	334 patients	US + Gamma imaging	73.70
**Proposed Model (DeiT-MLP-Mixer)**	**DeiT for global feature extraction + MLP-Mixer-inspired classification layer**	**502 patients (1506 augmented images)**	**Grayscale ultrasound**	**95.70**

As noted in the Related Works section, many previous methods were based on CNNs or radiomics models, which focused mainly on local features and often needed large labeled datasets. Some multimodal methods achieve better accuracy, but they require more complex data and are challenging to apply in practice. Our proposed model was designed to address these issues. By using DeiT to capture global features together and combining them with a simple MLP-Mixer classifier, we achieve an accuracy of 95.7%, even with a relatively small real-world dataset. This finding shows that our approach fills the gaps of previous methods and offers a more practical and effective way to predict ALN status before surgery.

**Fig. 8:**
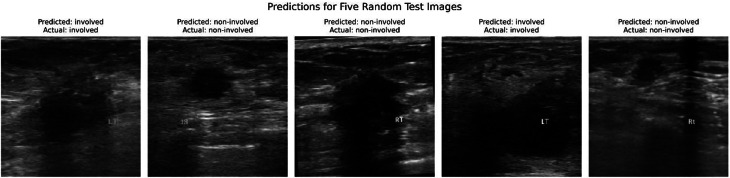
Prediction of the model from ultrasound images

Figure 8 shows the model’s ability to classify ALN involvement on five randomly selected ultrasound test images. Each image displays both the model’s prediction and the actual label. As shown, the first and fourth images are correctly identified as involved, whereas the second, third, and fifth images are correctly classified as noninvolved. This consistent performance highlights the model’s reliability and effectiveness in accurately determining lymph node status, which is crucial for clinical decision-making before surgery.

## Discussion

In this study, we developed a hybrid deep learning model called DeiT-MLP-Mixer to predict ALN status in breast cancer patients before surgery via grayscale ultrasound images. Our model reached 95.7% accuracy, along with strong precision, recall, specificity, and F1 score, while keeping the test loss low. These findings demonstrate that the model can reliably identify which patients have ALN involvement, even with a relatively small real-world dataset. The high specificity (0.987) and slightly lower, but still strong, sensitivity (0.924) reflects a deliberate trade-off in threshold-based classification. In the clinical setting of preoperative ALN prediction, high specificity is especially important because it reduces false positives that could lead to unnecessary procedures. At the same time, good sensitivity is essential to avoid missing cases with metastasis. Our model performs well compared with other noninvasive approaches, but we recognize that different clinical goals may require adjusting the threshold. In practice, the decision threshold can be changed to increase sensitivity at the cost of specificity, making the model more flexible as a tool for screening or triage. Future work will look at optimizing these thresholds to better match the model’s behavior to clinical risk priorities.

The model showed excellent probability calibration, with a low Brier score of 0.0378. The reliability diagram confirms that the predicted probabilities closely match actual clinical outcomes. At the clinical operating point, the model also demonstrated strong discrimination: when set to a high sensitivity of 95.0% for preoperative screening, it still achieved a specificity of 95.29%. This indicates a clear separation between classes, allowing the model to identify most positive cases without generating many false positives.

When we compared different models, ViTs presented clear advantages over traditional CNNs such as ResNet and VGG-16. ViTs can capture global patterns across image patches, which helps the model handle differences in lymph node appearance between patients. On the other hand, CNNs focus more on local details and struggle to combine larger pictures, which limits their performance on our dataset. We also compared ViT with DeiT, a more efficient version. DeiT performed better because it trains more effectively on small datasets and can adapt to subtle differences in ultrasound images. To make classification even more effective, we added an MLP-Mixer as the final classifier, creating the DeiT-MLP-Mixer. This head combines information from different parts of the image, making the model faster, more reliable, and better at picking up subtle patterns.

An analysis of misclassified cases showed that most errors occurred in borderline ALNs with unclear ultrasound features. False-positive predictions were often related to reactive lymph nodes with mild cortical thickening or uneven echogenicity, while false-negative cases typically involved nodes with micro-metastatic disease or a partially preserved fatty hilum. These situations reflect real-world clinical challenges, as the visual differences between involved and noninvolved lymph nodes can be subtle even for experienced radiologists. The model’s lower confidence in these cases likely reflects this natural overlap in ultrasound appearance rather than a systematic flaw, pointing to the potential value of additional imaging information or uncertainty-aware decision support in future work.

Although formal interpretability analysis was not performed in this study, the model likely relies on established ultrasound characteristics associated with nodal involvement, such as cortical thickness, hilum visibility, nodal shape irregularity, and internal echogenic patterns. These features are routinely evaluated by radiologists during preoperative assessment. Given that the DeiT backbone processes images as patches with global context awareness, it is plausible that the model integrates both local cortical features and broader structural patterns across the lymph node. Future work incorporating visualization techniques such as Grad-CAM or attention mapping could provide more explicit insight into which regions most influence the model’s predictions and further enhance clinical interpretability.

Compared with previous studies, our model shows clear improvements. Many past methods have relied on CNNs or radiomics, which focus on local features and often need large labeled datasets. Some multimodal approaches perform well but require more complex imaging data and are harder to apply in practice. By combining DeiT for global feature extraction with a simple MLP-Mixer classifier, our model achieves a high accuracy of 95.7% when only grayscale ultrasound images are used. This makes it practical, noninvasive, and easy to use in real clinical settings, with gaps left by earlier methods being filled.

From a clinical perspective, this model provides a noninvasive alternative to procedures such as SLNB and ALND, which can cause complications such as lymphedema or infection in up to 30% of patients [50]. Accurate predictions with the DeiT-MLP-Mixer could help reduce unnecessary surgeries, lower healthcare costs, and improve patient care. Its modest computing requirements also mean that it could be used in clinics with standard hardware, which is important for hospitals with limited resources.

The primary objective of the proposed system is to support treatment decision-making by providing early information on ALN status, which may influence the selection of systemic therapies such as chemotherapy or radiotherapy. In the longer term, reliable preoperative confirmation of lymph node negativity using AI–based approaches could support more conservative surgical planning and help reduce unnecessary invasive axillary procedures.

Despite these promising results, there are several limitations. The dataset was collected from a single clinic, so the model might not be generalizable perfectly to other hospitals or ultrasound machines. We acknowledge that the single-center design and limited size of the dataset (502 patients) are the main limitations affecting external generalizability. To reduce the risk of overfitting associated with the relatively limited dataset size, several regularization strategies were employed, including data augmentation, weight decay, early stopping, and learning rate scheduling. During pilot experiments, removing early stopping or increasing the learning rate resulted in less stable validation performance, suggesting that the model’s accuracy is influenced by appropriate regularization and fine-tuning settings. While the current configuration provided stable convergence, further large-scale studies could better assess sensitivity to hyperparameter selection. The DeiT architecture helps reduce the large data requirements typically associated with ViTs, but the proposed approach still needs to be validated on independent, multi-institutional datasets before it can be considered for clinical use. Differences in ultrasound machines, imaging protocols, and operator-dependent factors between institutions may influence image characteristics and potentially affect model performance. Ultrasound images are naturally affected by artifacts such as speckle noise, acoustic shadowing, and operator-related variations. Because the dataset was collected under routine clinical conditions, these variations were inherently present in both the training and testing images. We deliberately avoided applying manual denoising to preserve subtle texture details that may be clinically meaningful. In addition, data augmentation techniques were applied to simulate realistic acquisition variability and improve generalization. However, the model was not specifically evaluated under controlled artifact conditions. Further validation on multi-center datasets and under varying imaging settings will be important to confirm the model’s robustness and generalizability. We are currently exploring collaborative efforts to evaluate the model using data from public sources or collaborating hospital. Testing on data from multiple clinics or adding other imaging types, such as Doppler or elastography, could make the model more reliable. The current model has not been evaluated on cases with multiple small tumors, which remains an important area for future investigation. In addition, information regarding specific breast cancer molecular subtypes was not available in the collected dataset; therefore, subtype-specific performance was not assessed in this study. We also did not explore in detail how the model makes its decisions; tools such as Grad-CAM could help clinicians see which areas of the image the model uses, improving trust and interpretability. Future studies should include more challenging or unclear cases to fully test the model’s robustness.

There are several potential future directions. Researchers could test the model on larger, multi-institutional datasets and across different molecular subtypes to improve generalizability. Adding other imaging modalities or developing hybrid architectures that balance accuracy and efficiency could further enhance performance. In this study, we focused only on grayscale ultrasound images and did not include additional clinical variables such as patient age, tumor size, or molecular subtype, as these structured data were not available in the collected dataset. While this approach demonstrates the standalone capability of the proposed DeiT–MLP-Mixer, combining imaging features with relevant clinical information could further improve performance and model stability. Clinical and imaging data often provide complementary insights, and their integration may help achieve more comprehensive and personalized risk assessment. Exploring such multimodal approaches will be an important direction for future research using larger and more diverse datasets. Evaluating the model in real-time clinical workflows and improving interpretability will also be key for practical use.

Overall, the strong performance of DeiT-MLP-Mixer shows the value of combining global feature extraction with efficient classification. DeiT allows the model to see patterns across the whole image, whereas the MLP-Mixer efficiently combines information from different regions. This combination allows the model to detect subtle differences in lymph nodes that vary between patients, making it accurate, fast, and practical for clinical use. These findings suggest that while DeiT-MLP-Mixer effectively captures both global and local patterns in most cases, incorporating additional ultrasound information such as Doppler or elastography could further improve performance in borderline lymph node cases.

## Conclusion

This study revealed that the DeiT-MLP-Mixer can help specialists make better preoperative decisions by accurately predicting ALN status from grayscale ultrasound images. By combining transformer-based feature extraction with MLP-Mixer’s efficient classification, the model can capture both broad patterns and subtle details in images. This hybrid design could serve as a practical tool for clinical decision-making, even when data are limited.

Our results suggest that combining different deep learning strategies can improve both the accuracy and usefulness of ALN prediction compared with traditional methods. By combining global image understanding with efficient classification, the model offers a new and effective approach for preoperative assessment.

We also note several limitations. The dataset came from a single clinic, which may affect how well the model works for other patient populations or imaging systems. Differences in image quality and patient factors could also affect the results. Future studies should test the model on more diverse datasets and use techniques that help explain its predictions, making it easier for clinicians to trust and understand.

Future work should focus on validating the model on external and multi-center datasets to ensure generalizability, assessing its integration into clinical workflows, and making the model’s predictions more transparent and trustworthy for clinicians.

In summary, our study highlights the potential of the DeiT–MLP-Mixer to support breast cancer care. Further research can test its reliability in different clinical settings and explore ways to include it in routine workflows, providing specialists with a reliable and practical AI tool to help guide treatment decisions.

## Data Availability

The datasets used and/or analyzed during the current study are available from the corresponding author upon reasonable request. Practical conditions for access include submitting a formal research protocol that outlines the intended use of the data, along with a signed data-use agreement ensuring that the data will not be re-identified or used for commercial purposes.

## Abbreviations

AI: Artificial Intelligence
ALN: Axillary Lymph Node
ALND: Axillary Lymph Node Dissection
CAP: Computer-Aided Prediction
CNN: Convolutional Neural Network
CLS: Classification
DIST: Distillation
DeiT: Data-efficient Image Transformer
FN: False Negative
FP: False Positive
MLP: Multilayer Perceptron
SLNB: Sentinel Lymph Node Biopsy
SVM: Support Vector Machine
TN: True Negative
TP: True Positive
ViT: Vision Transformer
